# Two Proximal Skin Electrodes — A Respiration Rate Body Sensor

**DOI:** 10.3390/s121013813

**Published:** 2012-10-15

**Authors:** Roman Trobec, Aleksandra Rashkovska, Viktor Avbelj

**Affiliations:** Jožef Stefan Institute, Jamova 39, 1000 Ljubljana, Slovenia; E-Mails: aleksandra.rashkovska@ijs.si (A.R.); viktor.avbelj@ijs.si (V.A.)

**Keywords:** ECG-Derived, respiration, body, sensor, skin electrode, wireless

## Abstract

We propose a new body sensor for extracting the respiration rate based on the amplitude changes in the body surface potential differences between two proximal body electrodes. The sensor could be designed as a plaster-like reusable unit that can be easily fixed onto the surface of the body. It could be equipped either with a sufficiently large memory for storing the measured data or with a low-power radio system that can transmit the measured data to a gateway for further processing. We explore the influence of the sensor's position on the quality of the extracted results using multi-channel ECG measurements and considering all the pairs of two neighboring electrodes as potential respiration-rate sensors. The analysis of the clinical measurements, which also include reference thermistor-based respiration signals, shows that the proposed approach is a viable option for monitoring the respiration frequency and for a rough classification of breathing types. The obtained results were evaluated on a wireless prototype of a respiration body sensor. We indicate the best positions for the respiration body sensor and prove that a single sensor for body surface potential difference on proximal skin electrodes can be used for combined measurements of respiratory and cardiac activities.

## 1. Introduction

Breathing is one of the most obvious signs of human vitality and activity; however, it can also reflect the status of a patient and the progression of an illness. The entire process, from the inhalation to the exhalation, is referred to as breathing or respiration cycle (RC). The respiratory rate indicates the frequency of breathing or the time between two consecutive RCs. Any alterations in the respiratory rate can help predict potentially serious clinical events, such as a cardiac arrest, or it may suggest that a patient is admitted to an intensive-care unit [1].

Several techniques are available for measuring respiration-related signals. Some of them are noncontact [2] and therefore potentially advantageous for patient comfort; however, they require complex monitoring instruments, e.g., IR cameras. Additionally, a calibrated position of the patients is needed, which is not acceptable for sensor-like respiration monitoring. There have been several studies reporting developments in respiration monitoring. A comprehensive review of non-invasive respiratory monitoring in medical care can be found in [3]. The monitoring methods are categorized into those responding to movement, intrathoracic pressure, volume and tissue composition changes, air-flow velocity, or temperature and blood-gas concentration. The authors concluded that non-invasive respiratory monitoring is still in its exploratory phase, with numerous reports on specific device solutions, but less work on evaluation and adaptation to clinical requirements.

The ECG-derived respiration (EDR) techniques that are more relevant to our work are based on the observation that the positions of the ECG electrodes on the chest surface move relative to the heart. Additionally, the transthoracic impedance varies as the lungs fill and empty. A popular technique involves measuring the transthoracic impedance using these ECG electrodes [4]. The method requires special-purpose hardware and is not suited for recovering the respiration from a recorded ECG signal, but its significant advantage is that a continuous respiration-related signal is obtained. Another well-known method is based on observing the beat-to-beat variations in the duration of the RR intervals [5]. The RR variability occurs primarily due to respiratory sinus arrhythmia (RSA), particularly in young and healthy subjects, which is one of the limitations of this method.

A single-lead wireless approach, based on the EDR algorithm that measures the beat-to-beat amplitude variation of the QRS complex for infant hearts, was published in [6], but with no detailed clinical evaluation. There are also several later studies of EDR algorithms based on the amplitude variations of the ECG waves [7] that are associated with the respiratory-induced variation in the thoracic impedance and in the relative position of the ECG electrodes and the heart. Principal component analysis has been used to identify the most effective ECG lead before extracting the respiration rate [8]. This method is particularly effective if two or more ECG signals are available. The EDR can be obtained even in patients with congestive heart failure, where RSA may be absent. In [9] a quantitative comparison of the EDR monitoring techniques based on a direct measurement of the modulation components *versus* techniques based on the calculation of the mean electrical axis variation was carried out. The authors concluded that single-lead respiration-rate estimates are more robust than the methods based on the mean electrical axis. In [10], different EDR principles, *i.e.*, electrical impedance pneumography across the chest, a photoplethysmogram across a finger, and an ECG signal, were fused to achieve more robust results. The estimation of the breathing rate was still limited by artifacts introduced by movements. Thirteen different algorithms for detection of the sleep apnoea from ECG recordings were analyzed in [11]. The best algorithms made use of the frequency-domain features to estimate the changes in the heart rate and the effect of respiration on the ECG waveform. The respiration-related heart-rate variability is reduced in the elderly, but the ECG waveform variability persists, regardless of age; so, the latter could have a greater overall potential.

Most of the above-mentioned approaches for the extraction of the respiration signal are based on wired electrodes and external devices that cannot be considered as body sensors. We have prototyped a differential wireless bio-electrode (WBE) for measuring surface potential differences [12], initially intended to act in a set of three body electrodes that are able to synthesize the standard twelve-lead ECG [13]. We recognized that other features can also be added to such an electrode, e.g., the measurements of the vessel pulse [14], the skin resistance and the respiration-rate. If a multi-function body sensor is able to act as a wireless unit, it can represent an important technological breakthrough. For example, the development of Telemedicine/Telecare systems can benefit from the potential non-obstructiveness, simplicity, and reusability of such sensors [15].

The number of body sensors should be as small as possible, because a smaller number of sensors is less disruptive for users. Therefore, an important aspect in the design of new body sensors should be multi-functionality, *i.e.*, several vital indicators should be extracted from a single monitored bio-signal. For example, body surface potentials are normally used for monitoring heart (ECG), brain (EEG), muscular contraction or respiration activity.

The second important aspect, related to the usability of the body surface potential, is the proximity of electrodes. If the potential is measured from proximal skin electrodes that have to be electrically connected, we obtain a small plaster-like body sensor that is able to measure a differential body surface potential. The proximity of the electrodes is very important in the context of body sensors because users have to consider both electrodes as a single unit.

The last aspect of multi-function body sensors is their ability to act as wireless units, which substantially improves their usability and acceptability for the users. However, a lot of work remains to be done in order to select the correct balance between the ability for local signal processing, the complexity of the communication protocols, and the autonomy of the power supply. In this paper, we will concentrate on the extraction of the respiration signal from the potential difference of two proximal body surface electrodes, approximately 5 cm apart, which act as a single multi-function body sensor. We propose a multi-function sensor design and evaluate its applicability using clinically recorded 35-channel ECG measurements with a simultaneously recorded respiration signal obtained from a nasal thermistor. We identify the best positions for the respiratory sensors and confirm that the proposed approach is a viable option for wireless monitoring of the respiration rate.

The rest of the paper is organized as follows: in the next section, the methodology for the evaluation of the best positioning of the respiration sensor based on the analysis of 91 potential differences from the proximal electrodes of 35-channel ECG is described. A prototype design of a multi-purpose body sensor, currently applicable for measurements of the respiration and the cardiac activity, is presented. The performances of the respiration sensor are evaluated by real measurements of the respiration signal from a nasal thermistor. In Section 3, the analyses of 15 measurements from seven healthy volunteers are presented. The best positions of the respiration body sensor are identified. In the Discussion, the advantages and difficulties of the proposed methodology are analyzed. The paper concludes with a summary of our findings and potential future extensions of the proposed multi-purpose body-sensor approach.

## 2. Methodology

Here, we explore the quality of the EDR in dependence of the sensor's position using a multi-channel ECG (MECG) and considering all the pairs of two neighboring electrodes as virtual respiration sensors. The results are evaluated using a reference respiration signal obtained from a measurement of the air temperature near the nose and mouth using a thermistor. If the temperature of the thermistor increases, because of the expired air, its resistance decreases and consequently the respiration signal is diminished. Furthermore, we developed a prototype of a wireless multi-function sensor with implemented EDR algorithms.

### 2.1. Study Population

Seven healthy volunteers with no previous medical record related to heart disease and with normal 12-lead ECGs were included as subjects in the study. Informed consent was obtained from all the subjects before the study. The clinical status, age, and gender are shown in Table 1. Two MECG measurements during spontaneous breathing were taken from each volunteer. The first measurement was taken after 15 minutes of resting for a period of 360 seconds during continued resting. The second measurement was taken after 5 minutes pause in the same way as the first measurement. Three measurements, each 250 seconds long, were obtained only from volunteer no. 7. All the recorded data were immediately examined for the quality of the signals from each individual electrode. In the case of a defective measurement, e.g., a bad contact of an electrode, the cause of the disturbance was solved and the data acquisition was restarted. The recorded data were saved for further processing. No attempt was made to exclude any measurements; therefore, a few measurements have seldom arrhythmic events. All the measurements were obtained during our study at the University Clinical Centre Ljubljana and the Jožef Stefan Institute.

### 2.2. MECG Measurements

**Figure 1.**
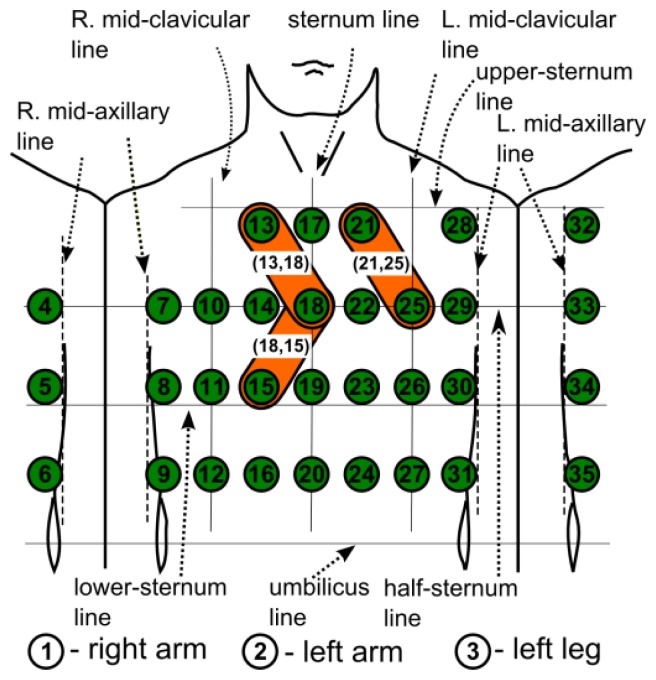
Schematic locations and numbering of 35-channel MECG electrodes on the chest. Potential differences between neighboring electrodes, e.g., (13,18), (21,25), (18,15), (14,19), (22,23), (31,35), *etc.*, are denoted as differential leads (DLs).

MECG measurements with 35 leads, all referenced to the Wilson central terminal potential (see [16] for details), were used in our analysis. From a subset of the MECG leads {1, 2, 3, 14, 18, 23, 26, 31, 35} a standard 12-lead ECG was generated as: I = 2 − 1, II = 3 − 1, III = 3 − 2, aVR = −(I + II)/2, aVL = I − II/2, aVF = II − I/2, V1 = 14, V2 = 18, V3 = 23, V4 = 26, V5 = 31, V6 = 35. The electrode positions are adapted to the body size, and consequently the distances between the electrodes differ slightly for different subjects. The locations of the electrodes are shown schematically in Figure 1. The MECG device also incorporates one additional channel for the simultaneous measurement of the thermistor signal. The measured analog signals are sampled at 1,000 Hz and digitized with a 0.73 μV resolution (14-bit analogue-digital converter). The bandwidth of the recording system is 0.05 to 250 Hz.

The recorded signals were imported and further processed using MatLab (MathWorks, Inc.). A low-pass filter (cutoff frequency 40 Hz) was used on all the measurements with attenuation of 60 dB and stop frequency of 100 Hz. The baseline wandering was removed with a two-pass median filter of 200 ms and 600 ms window widths to remove the PQRS complexes and T-waves. The signal resulting from the second pass contains the ECG baseline, which is then subtracted from the original signal to produce an ECG signal with a removed baseline. We denote each MECG measurement by a set of leads as:

(1) X={X(1),…,X(i),…,X(j),…,X(n)}

where x(i) and x(j) are the i-th and j-th leads, and n is the total number of leads, in our case 35. We use MECG measurements as a data source for emulation of the bipolar measurements that can be obtained by the body surface potential sensor. For the 35 leads of an MECG there are $\left(\begin{array}{c} 35\\ 2 \end{array}\right)=595$ possible differences between them. However, the implementation of a small respiration body sensor dictates a minimization of the distance between pairs of MECG electrodes. This restricts the set of possible pairs to only the nearest neighbors, referred to as differential leads (DL):

(2) $$\begin{array}{cc} \text{DL}k=X(i)-X(j), & i,j=1,\ldots ,35\;\text{and}\;k=1,\ldots ,91 \end{array}$$

where pairs of i and j are chosen so that only the nearest-neighboring leads are taken into account. This results in 91 DLs. A shorter notation for a DL is (*i*,*j*). For example, referring to Figure 1, the three DLs that are marked by a plaster-like area encompassing the electrode pairs, (13,18), (21,25), (18,15), are DLs, *etc.*, but the electrode pairs (18,25), (11,26), *etc.*, are not. Such an approach results in 91 DLs that emulate respiration sensors at specific positions on the body surface. The minimal inter-electrode distance remains 5 cm, which ensures an acceptable S/N ratio [17].

To have a more compact representation of the results, we marked the DLs alternatively with consecutive numbers. We start at electrode X(13) and always consider first the right neighbor in the same row, *i.e.*, *X*(17), to obtain DL1 = *X*(13) – *X*(17). We then consider the neighboring electrodes from the row below, *i.e.*, *X*(10), *X*(14), *X*(18), to obtain DL2 = *X*(13) – *X*(10), DL3 = *X*(13) – *X*(14), and DL4 = *X*(13) – *X*(18), and so on until the final DL91 = *X*(31) – *X*(35). The DLs from Figure 1: (13,18), (21,25), (18,15), (14,19), (22,23), (31,35) can be shortly denoted as: 4, 11, 34, 32, 39, 91.

### 2.3. ECG-Derived Respiration

**Figure 2.**
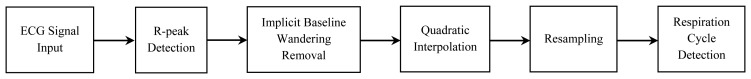
Block diagram of the implemented EDR algorithm.

The block diagram of the implemented EDR algorithm is shown in Figure 2.

The positive short peaks (usually R peaks) in the ECG signal are determined with an adaptive detector that runs on the first derivative of the ECG signals. The first derivative is obtained by calculating the differences between each two neighboring samples. The peak detector searches for four consecutive positive and four consecutive negative derivatives above a threshold in a time window determined by the expected peak width. If such an event occurs, it is declared as R-peak. The threshold values are estimated dynamically as 10% of the average from the maximum and minimum calculated derivatives. The search window is 50 ms. The maximum value of R-peak (MR) inside the current interval is found, and anytime that a peak is found, the search counter is increased by the width of the search window to jump over the detected peak. It is not unusual for negative peaks to be present in the MECG channels; therefore, we also repeat the above procedure on inverted measurements to find the eventual negative peaks.

The collected MR values are in fact the respiration signal “sampled” at R-peak times. We reconstruct the respiration signal using the ECG signal with removed base-line wandering. This means that the values of the respiration signal are now the same as the MR values. Such an approach is quite demanding, because the baseline-correction algorithm has to be implemented before the reconstruction of the respiration signal.

We also tested the approach with the raw ECG signal as input. The values of the respiration signal were obtained as the differences between the MR values and the average ECG signal amplitudes in a 20-ms window positioned in the middle of the P-Q interval. In all cases, the middle of the P-Q interval was positioned 100 ms before the R-peak time. Such an approach implicitly removes the base-line wandering and is therefore less computationally demanding. The number of detected RCs differs slightly in just a few cases compared to the first approach where the base-line wandering is previously removed. Therefore, we used the second approach, which might be more appropriate in real implementations.

Theoretically, the respiration can be derived from the variations in the amplitudes of the ECG signal if the heart rate is at least two times greater than the respiration rate, which is often the case. We interpolate the R-peak amplitudes using a quadratic polynomial and resample the signal with two-times higher frequency than the average heart rate. The first derivative of the re-sampled signal is calculated based on the differences between neighboring samples. Then, a RC detector is applied. It searches for two or more consecutive positive derivatives followed by two or more consecutive negative derivatives, which indicates a new RC. Again, when an RC is identified, a new RC search begins, starting from the end of the last detected RC.

The most frequently detected number of R-peaks, provided by a software-implemented R-peak detector that runs on all the available leads, is declared as the reference number of R-peaks (RefR). A visual inspection of a lead with RefR followed in order to confirm the number. No manual intervention was needed. The reference number of RCs (RefRC) is obtained from a visual inspection with counting the RCs on the thermistor signal.

The quality of a lead is defined as the average frequency of a correct identification of RCs in a set of analyzed measurements. An ideal lead will be able to correctly identify all the RCs in all the measurements. Because of uncontrolled breathing that incorporates the natural differences between the tested subject, and because of the inability of the RC detector to adapt to all these specifically, we allow a small number of falsely detected or undetected RCs. In our investigation, we insert all the leads with an exact identification of all the RCs in list RC0; identification lead lists with one, two or three erroneously identified or missed RCs denoted as RC1, RC2 and RC3, respectively.

Some leads are characterized by simultaneous variations of the positive and negative amplitudes that are pronounced enough for a successful identification of the RCs. For this reason, such leads could appear two times in the RC lists, e.g., in RC0 and RC1, if such a lead detects the exact number of RCs from the positive amplitudes and only a single error is made when detecting from the negative amplitudes. For the above example, we count only the best result in RC0 and remove the lead, which was successful twice, from the RC1 list.

### 2.4. Respiration Body-Sensor Prototype

**Figure 3.**
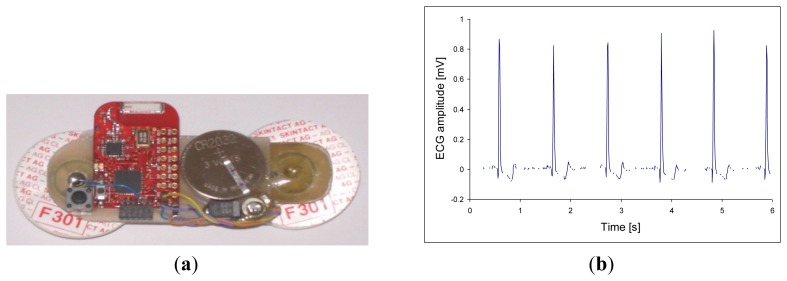
(**a**) Prototype of wireless respiration sensor with two, self-adhesive, disposable electrodes, a lithium coin battery, and a ceramic chip antenna. (**b**) Raw signal from the respiration sensor with ECG R-peaks modulated by breathing.

Body sensors should be non-disruptive to the users; therefore, we considered small and multi-functional wireless sensors. We prototyped a differential WBE for measuring the surface potential and differential synthesized ECG [12,13]. The same device can be used as a respiration sensor based on the EDR technique. Other features can also be added to this body sensor: EEG, vascular pressure, skin resistance and respiratory-rate measurements. Such an electrode will represent an important worldwide technological breakthrough. An example of the prototype multi-sensor electrode is shown in Figure 3(a); while Figure 3(b) shows its measured raw ECG signal. A respiration signal as an envelope of the R-peaks is clearly visible.

## 3. Results

**Figure 4.**
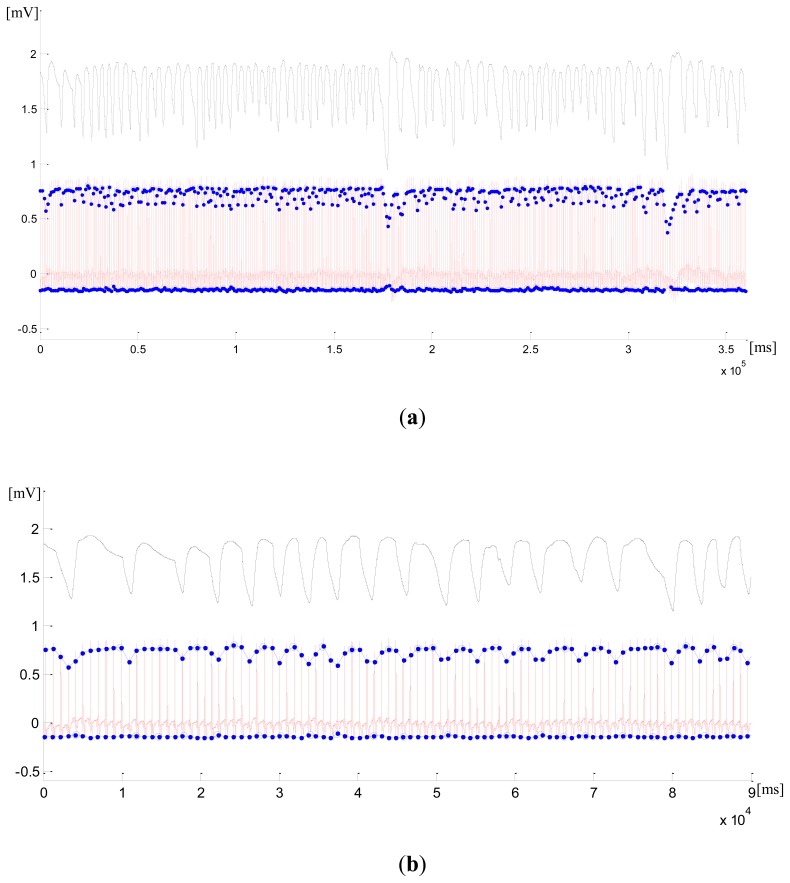

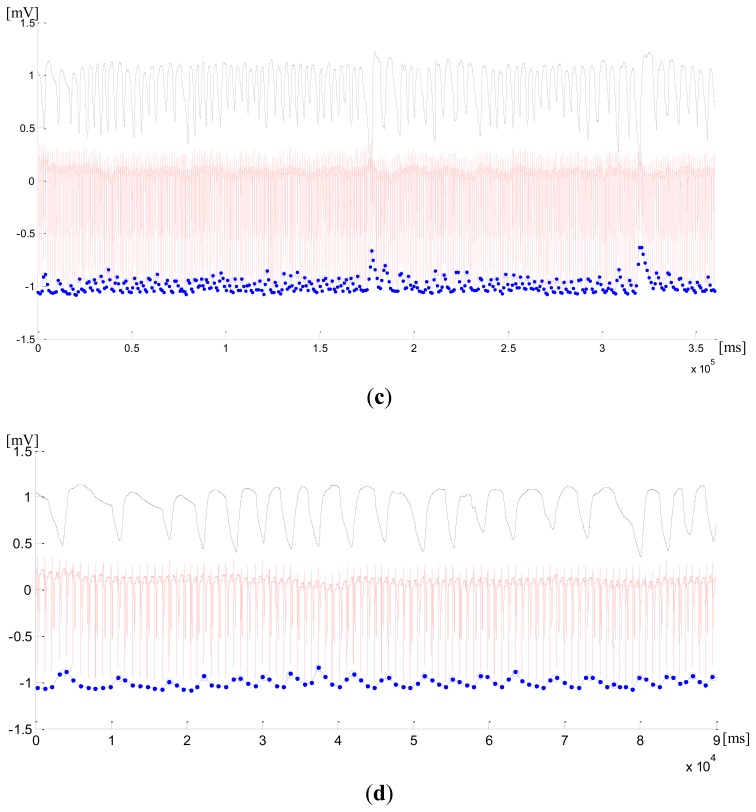
First measurement from the first subject case. (**a**) thermistor signal (black) with 78 RCs and (21,18) signal (red) with 385 R-peaks (blue dots) and 79 identified RCs (blue circles). (**b**) Zoomed section of the initial 90 seconds interval in (21,18). (**c**) (21,25) with 385 R-peaks and 79 identified RCs. (**d**) Zoomed section of the initial 90 seconds interval in (21,25).

The signal from the first measurement for the first subject case, measured by DL (21,18) (continuous signal in red), with significant variations in the positive amplitude, is shown in Figure 4(a). The blue dots on the signal amplitude envelope mark 385 detected R-peaks and the blue circles mark 79 detected RCs. The thermistor signal (black) shows 78 RCs, which are taken as RefRC. We can see from the zoomed initial interval of 90 seconds in Figure 4(b) that the very first RC is not detected because of the lack of a recorded signal. We further see a falsely detected RC after 50 seconds from the start of the measurement. Even though the variations in the negative amplitudes on (21,18) are very small, they still enable the detection of RCs.

The negative peaks in the respiration signal are shown to be considerably pronounced. They can be detected in (21,25) with a significant variation in the negative amplitudes. Here, all the R-peaks have been identified correctly, but again we have 79 detected RCs with one falsely detected RC at 77 seconds. The error happens during expiration, which was not smooth, but stalled for a while. We can see from the zoomed sections that, besides the respiration rate, we could also reconstruct the length and the shape of most of the RCs in the respiration signal. The exact classification of the RCs could improve their detection and identification, but this is beyond the scope of our paper.

**Figure 5.**
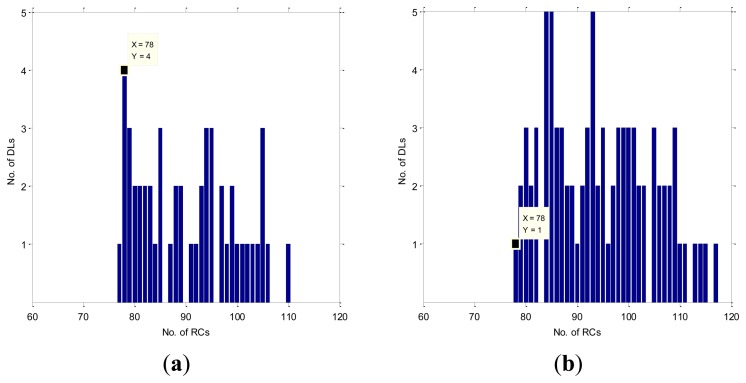
Bar plots of the number of detected RCs from the first measurement of the first subject case from (**a**) respiration signal in positive amplitudes for all DL and (**b**) respiration signal in negative amplitudes for all DLs.

However, the reconstruction of the respiration signal is not so successful on many of the remaining DLs. In Figure 5 the histograms of the reconstructed RCs are shown for the case SC1M1 and for all the DLs variations in their positive (a) and negative (b) amplitudes. The RefRC, which is 78, is marked with a label. We have four correctly identified numbers of RCs on DLs with variations in the positive amplitude {(13,18) (14,18) (25,29) (8,12)} and a single correctly identified RC on (30,27) with variations in the negative amplitude. Consequently, the RC0 list will have five elements. In the same way, we have four DLs with a single falsely detected or missed RC on positive amplitude variations and two DLs with negative amplitude variations, so the RC1 list will have six elements. We can read in the same way from Figure 5 that the RC2 and RC3 lists will have five and four elements, respectively.

Because of the many falsely detected RCs, e.g., in Figure 5 eight DLs have identified 85 RCs, we could not clearly decide about the exact number of RCs if we did not have the reference thermistor signal. Therefore, the evaluation of the identification quality is needed for each lead, which can improve the EDR reliability and robustness.

**Figure 6.**
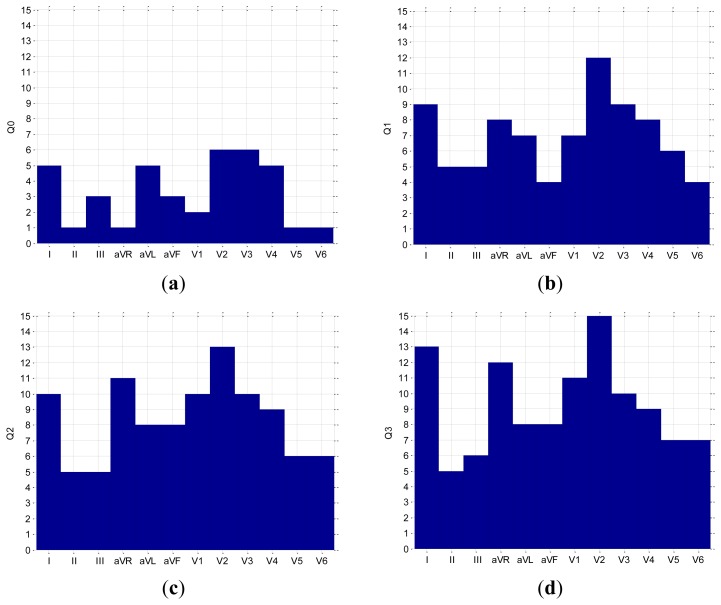
Bar plots of the lead quality (**a**) Q0, (**b**) Q1, (**c**) Q2 and (**d**) Q3 for all leads from a 12-lead ECG.

**Figure 7.**
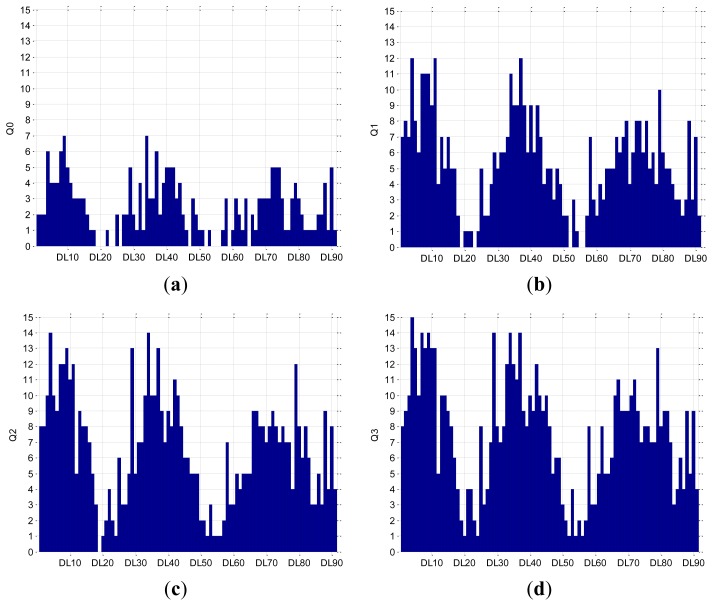
Bar plots of the lead quality (**a**) Q0, (**b**) Q1, (**c**) Q2 and (**d**) Q3 for all DLs.

The quality of the EDR for all the DLs and for the standard 12 leads was obtained from the analysis of measured results from all the subjects, as described in Section 2.3. The quality of the leads is calculated by counting the number of appearances of each lead in the lists RC0, RC1, RC2 and RC3. If a lead would be in the RC0 list for all measurements, then it would have a quality rating Q0 equal to the number of measurements. If we relax the requirements and allow up to two falsely detected RCs, then we consider the union (∪) of all the elements from the lists RC0 and RC1, because if a lead appears in RC0 and RC1 it should be counted only once. The quality rating *Q*1 = (*RC*0 ∪ *RC*1) indicates the identification reliability of ±1 RC per 360 seconds (duration of measurements). If we further release the requirements and allow up to four falsely detected RCs, then we obtain *Q*2 = (*RC*0 ∪ *RC*1 ∪ *RC*2) with an identification reliability of ±2 RCs per 360 seconds. Finally, the quality rating *Q*3 = (*RC*0 ∪ *RC*1 ∪ *RC*2) indicates the identification reliability of ±3 RCs per 360 seconds. We can consider Q0 and Q1 as exact, while Q2 and Q3 represent average relative errors of 2.5% and 5%, respectively. The assembled lead quality results are shown in Figure 6 for a 12-lead ECG, and in Figure 7 for all 91 DLs. We could select a particular DL and fix the respiration sensor from Figure 3 in its position, expecting the quality that is calculated using our methodology.

**Figure 8.**
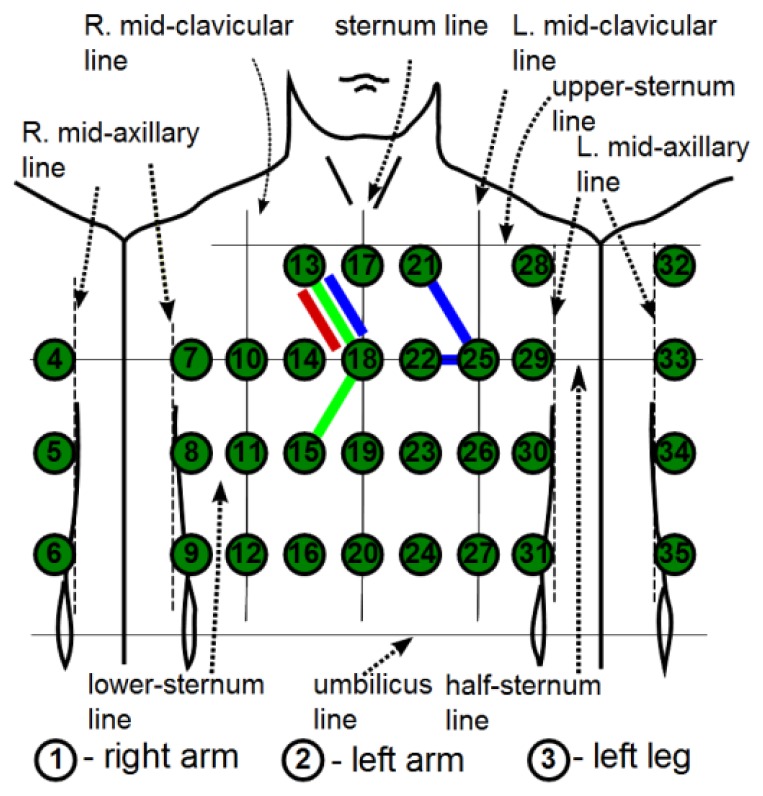
Schematic locations of the best DLs that correctly identify all RCs in 12 out of 15 measurements (blue), DLs with the relative error of 2.5% in RCs identification in 14 measurements (green), and DLs with the relative error of 5% in RCs identification in all 15 measurements (red).

We see from Figure 6(b) that the best lead in the 12-lead ECG is V2, with all correctly identified RCs in 12 out of 15 measurements, while Figure 7(b) shows that the best DLs are 4, 11 and 37, *i.e.*, (13,18) (21,25) (22,25), with correctly identified RCs in 12 measurements. If we allow a relative error of 2.5% (±2 RCs), then in the 12-lead ECG, lead V2 remains the best with correctly identified RCs in 13 measurements [Figure 6(c)], while in the DLs, the number of measurements increases to 14 in DL4 and DL34, *i.e.*, (13,18) (18,15) [Figure 7(c)]. If we further relax the relative error to 5% (±3 RCs), then in the 12-lead ECG, lead V2 [Figure 6(d)], and in DLs, DL4 [Figure 7(d)], succeed in all 15 measurements. However, five alternative DLs: 7, 9, 29, 34, 37, *i.e.*, (17,18) (21,18) (14,18) (18,15) (22,25) were successful in 14 measurements. The results for DLs are schematically presented in Figure 8.

## 4. Discussion

In this work, we confirm that DLs can also be successfully applied in the derivation of the respiration signal. Moreover, with the approach based on DLs, we can implement a multi-function body sensor that can be used simultaneously for a reliable synthesis of the standard 12-lead ECG and for derivation of the respiration rate.

Based on our analysis, the DLs proposed in Figure 8 are considered to be the most suitable ones for EDR. We have determined the DLs that correctly identify all RCs in most of the subject cases. Also, we have determined the DLs with a relative error of 2.5% and 5% in RCs identification, but still applicable for EDR with a satisfying accuracy. However, results indicate some variations among subjects in the ability of the DLs for EDR. This effect is related to the fact that the respiration induced R wave modulation is caused by the movement of the heart relative to the electrical axis of the selected DL, and that the respiration mode (diaphragmatic *versus* thorax breathing) and electrical axis differ among the subjects [18]. However, we confirmed that the presented methodology provides consistent and repetitive results; therefore, further measurements and validations are not needed for every subject.

We confirm that several DLs on positions near the center of the chest provide adequate signals for EDR algorithms that can reliably extract respiration rates from variations in R-peak amplitudes. The proposed methodology is accurate enough for most practical cases and therefore useful for mobile health (m-health) applications based on body sensors. Alternative methods, even if they use a dedicated respiration sensor, achieve the same level of accuracy [2], because the main difficulties lie in the correct identification of RCs, *i.e.*, the detection of peaks in the respiration signal, and not in the accuracy of the respiration signal recordings.

There are several potential advantages of the methodology used in the implementation of the respiration sensor. The potential between two proximal skin electrodes is often less sensitive to external electromagnetic influences and muscular noise. Eventual signal distortions in the standard bipolar leads from the extremities, which form the reference Wilson potential, have a small impact on the signal measured by the respiration sensor. We can adjust the position and the orientation of the body respiration sensors, which has a crucial impact on the detection accuracy.

The possible limitations of our research are the relatively small number of subjects and the fact that only healthy subjects were investigated. We did not experience a lot of arrhythmic events and other abnormalities in the ECG, which could potentially reduce the accuracy of the obtained results. However, if the respiration rate could be monitored with some admissible error, such events can be neutralized.

We found that the algorithms for the baseline correction and the R-peak detection are sufficiently accurate in various conditions and for different subjects. However, this is not the case in the peak detection of the EDR signals. In the case of fast breathing there are not enough samples (R-peaks) per RC. There are a lot of different types of breathing, specific for different subjects, e.g., prolonged, delayed, double, *etc.* Adaptive algorithms for the detection of respiration peaks should be developed in order to be appropriate for general use and high levels of accuracy.

Our work could be extended in several directions. The implementation of a multi-function body surface potential sensor could be improved by reducing the power consumption for a prolongation of its autonomy, by improving its design to become even less obstructive for users, by the sensing of other biosignals, e.g., skin resistance and temperature, muscle potential, vessel pulse, *etc.*, and also by the inclusion of more complex functionality, like the detection of unusual events. In particular, a new prototyped muscle-contraction (MC) sensor [14] is considered for integration into the system. The sensor is relatively small and light. It is based on a novel principle for measuring the muscle tension during muscle contractions and provides important data about the patient's muscular activities. Additionally, we consider the option to use the MC sensor for the detection of the tactile pulse.

## 5. Conclusions and Outlook

We evaluated the possibility of extracting the respiration signal from the potential difference of two proximal body surface electrodes that act as a single multi-function body sensor. We proposed a multi-function sensor design and evaluated its applicability using clinically recorded 35-channel ECG measurements with a simultaneously recorded respiration signal obtained from a nasal thermistor. The results were confirmed by a prototype wireless respiration sensor. We identified the best positions for the respiratory sensors and confirmed that the proposed approach is a viable option for wireless monitoring of the respiration rate. If MECG or wireless sensors are not available, the results can be reproduced using the limb electrodes (right arm – RA, left arm – LA, right leg – RL, left leg – LL) of a standard 12-lead ECG. For example, if LA, RA and LL are positioned as the MECG electrodes 13, 18 and 15, then the ECG lead I will be equivalent to DL (13,18) and the ECG lead II to DL (15,18).

A multi-function body sensor acting as a wireless unit can represent an important technological breakthrough because of its potential non-obstructiveness, simplicity and reusability. The multi-functionality could become an important aspect in the design of new body sensors because several vital functions can be extracted from a single monitored bio-signal. For example, the body surface potential indicates heart, brain, respiration, muscular, neural and other activities. The proximity of the electrodes is very important in the context of body sensors because users have to consider both electrodes as a single unit. The wireless implementation of the multi-function body sensor could substantially improve its usability, for example, in future Telemedicine/Telecare systems.

Future work is needed to improve the performance of the respiration sensor by reducing its power consumption to prolong its autonomy, and by improving its design to make it more acceptable to users. The local signal-processing algorithms can become adaptive and personalized, resulting in better reliability with a minimum of false alarms. The sensor's multi-functionality can be further extended by sensing other bio-signals.

## Figures and Tables

**Table 1.** Data for subjects included in the study.

**Clinical status**	**Number of subjects**	**Age (mean ± SD)**	**Gender (female:male)**
NPMR	7	45.6 ± 9.6	5:2

NPMR: no previous medical record.
